# Supplementary material for: Heatwave attribution based on reliable operational weather forecasts
Source: Nat Commun. 2024 May 30;15:4530. doi: 10.1038/s41467-024-48280-7 (PMC11140005; doi:10.1038/s41467-024-48280-7)
Supplement: Supplementary file 1 — Supplementary Information [file 41467_2024_48280_MOESM1_ESM.pdf]

# Supplementary information for *Reliable heatwave attribution based on successful operational weather forecasts*

Nicholas J. Leach,[1,2*] Christopher D. Roberts,[3] Matthias Aengenheyster,[1,3]
Daniel Heathcote,[1,4] Dann M. Mitchell,[4] Vikki Thompson,[4,5]
Tim Palmer,[1] Antje Weisheimer,[1,3,6] Myles R. Allen,[1,7]

[1]Atmospheric, Oceanic, and Planetary Physics, Department of Physics,
University of Oxford, Oxford OX1 3PU, UK
[2]Climate X Ltd., London, EC2N 2JA
[3]Earth System Predictability Section, Research Department,
European Centre for Medium-Range Weather Forecasts, Reading RG2 9AX, UK
[4]School of Geographical Sciences,
University of Bristol, Bristol BS8 1SS, UK
[5]Royal Netherlands Meteorological Institute (KNMI),
3731 GA De Bilt, The Netherlands
[6]National Centre for Atmospheric Science,
Atmospheric, Oceanic, and Planetary Physics, Department of Physics,
University of Oxford, Oxford OX1 3PU, UK
[7]Environmental Change Institute, School of Geography and the Environment,
University of Oxford, Oxford OX1 3QY, UK

*To whom correspondence should be addressed; E-mail: nicholas.leach@physics.ox.ac.uk.

# S1 Perfect-model experiment

In this section, we describe a perfect-model experiment carried out with the coupled slab ocean-atmosphere model HadSM4 that mirrors the design used in the main text. This model combines the atmosphere-land global model HadAM4[1,2,3,4,5] with a simple slab ocean model[6,2]. It is very similar to the configuration used by[7] who coupled a slab ocean to a lower-resolution HadAM3 configuration[8]. The atmosphere is run at N144 resolution (1.25° x 0.83°) with 38 vertical levels, of which 8 levels are in the boundary layer. At each atmospheric grid cell, the slab ocean is represented by a mixed layer with a constant thickness of 50 m that exchanges heat with the atmosphere through surface heat fluxes and the deep ocean by a provided ocean heat convergence, representing slow ocean forcing onto the mixed layer. With no vertical structure and a spatially and temporally constant mixed layer depth it is therefore consistent with previous work[2,7,9,10,11] (note that[12,13] use a smaller depth of 2.5 m for faster equilibration), but relatively simple compared to newer slab ocean models[14,15]. The slab ocean includes the sea ice model of HadCM3, with a simple treatment of dynamics where sea ice is advected by prescribed velocities ($u_{ice}$,$v_{ice}$). The timestep of $\Delta t_s$ = 1day does not resolve the diurnal cycle. Like HadAM4, HadSM4 is run on the volunteers computers as part of the climateprediction.net system[16,17,18]. It has much lower computational requirements than more recent Met Office models while achieving comparable performance for extratropical winter climate[3,4] and can thus generate many more ensemble members.

We use identical observationally-informed perturbations to the initial states of ocean temperature, sea ice concentration and sea ice thickness to those described in the methods section. The standard approach within climateprediction.net to generate initial condition ensembles is to perturb the initial 3D potential temperature at 38 vertical levels with sequential daily temperature differences taken from a control run. These perturbations are thus dynamically informed

by the attractor of the model (i.e. they are not white noise) and can be expected to sample the fastest-growing modes to a satisfactory degree. However, the perturbations tend to be quite large (up to 20 K), so the memory of the initial state is lost very quickly. This makes these perturbations unsuitable for predictability studies. Instead, for the experiments we performed here, the standard perturbations are scaled by a factor of 0.3, which was found to yield a comparable if slightly lower error growth compared to the ECMWF medium-range and S2S ensembles. This may be due to the lack of more advanced features of forecast models, such as stochastic physics and perturbations derived using data assimilation.

For this perfect-model experiment, we selected a "heatwave", with an estimated return period of 10 years, that occurred in a single model realisation in the first week of February. We then ran initial condition ensembles as described above at several different lead times, ranging from a few days to three months. We initialised a 500-member ensemble at each lead time for three scenarios representing current, pre-industrial and future climate states through the initial condition perturbations described above, plus perturbations to the $CO_2$ concentration. The results of these ensembles are shown in Figure S1. Summarising, these ensembles show that while the raw estimated attributable climate change signal within the heatwave is reduced for short lead times, due to the model adjustment to the new climate state, consistent estimates of this signal across lead times can be obtained by normalising the local response by the global land surface temperature response, as we did in the main text. This provides confidence in the approach taken to account for these changes to the attributable signal with lead time.

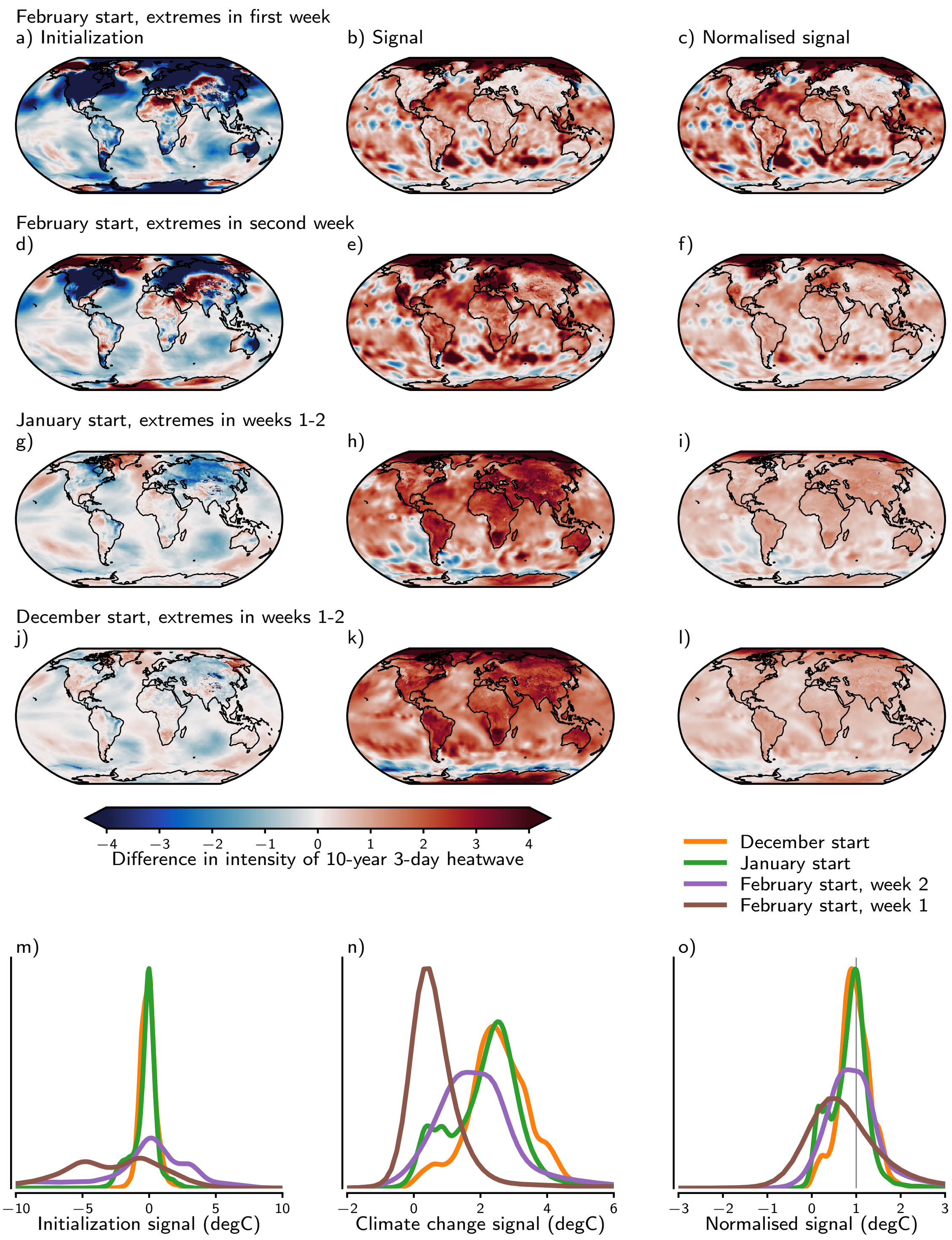


Figure S1: **Summary figure for HadSM4 perfect model experiment.** *Continued overleaf.*

Figure S1: **Panel a:** change in intensity between the current and climatological ensemble of an estimated 1-in-10-year extreme 3-day heatwave occuring in the first week of February for ensembles initialized on February 1st. This shows how different the the current ensemble is from climatology on lead times of less than a week, i.e. the role of initialization or the predictability on those timescales. **Panel b:** as **a** but for the change in intensity between the future and pre-industrial ensembles. This shows the climate change signal for a 10-year heatwave that has emerged on timescales of less than a week. **Panel c:** as **b** but showing the intensity change divided by the ensemble mean change in global land surface air temperature over the first week of February. **Panels d-f** as **a-c** but for events in the second week in February. Panels **g-i** as **a-c** but for events in the first two weeks of February and ensembles initialized on January 1st. **Panels j-l** as **a-c** but for events in the first two weeks of February and ensembles initialized on December 1st. **Panels m-o** aggregating **panels a-l** for global land points. **m** demonstrates the loss of initialization signal for longer lead times - the distribution collapses around zero. **n** shows the increasing climate change signal, which over land is still small in the first week and is very consistent for January initialization. The normalized signal in **o** shows that the magnitude of a 10-year heatwave increases closely in line with global surface land temperature.

# S2 Methods-supporting figures

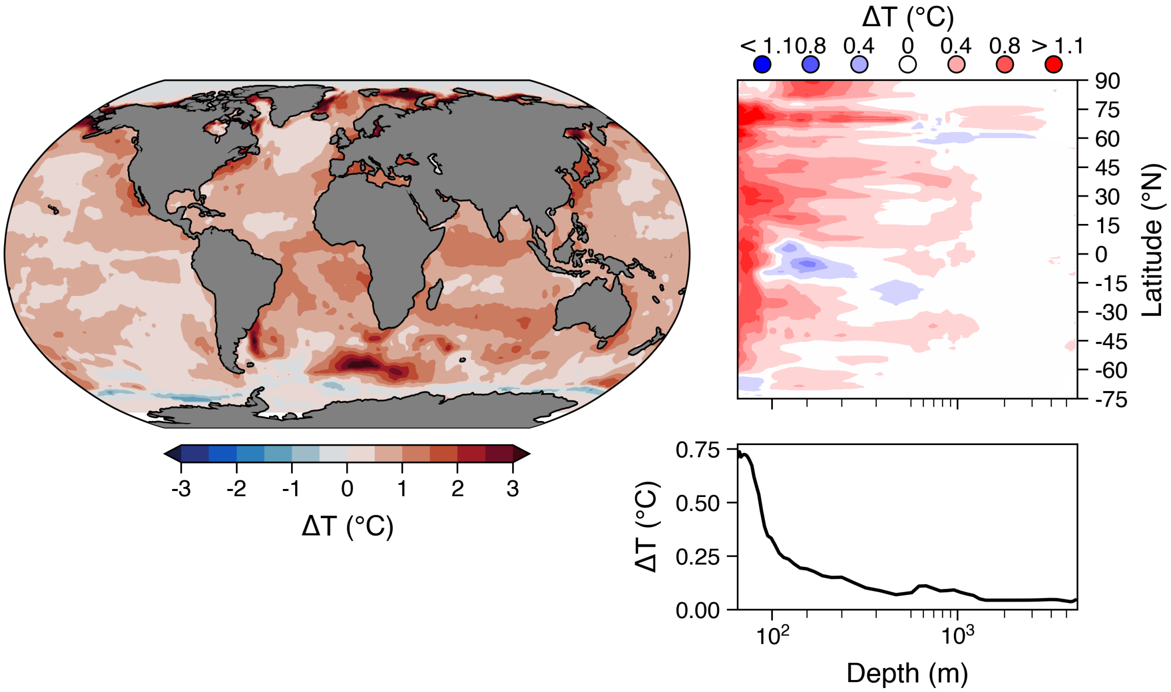


Figure S2: **The initial ocean state perturbation applied. Left panel:** map of the surface temperature perturbation. **Top right panel:** map of zonally averaged temperature perturbations as a function of depth. **Bottom right panel:** globally averaged temperature perturbation as a function of depth. Note that the x-axis switches from a linear to logarithmic scale at a depth of 500m.

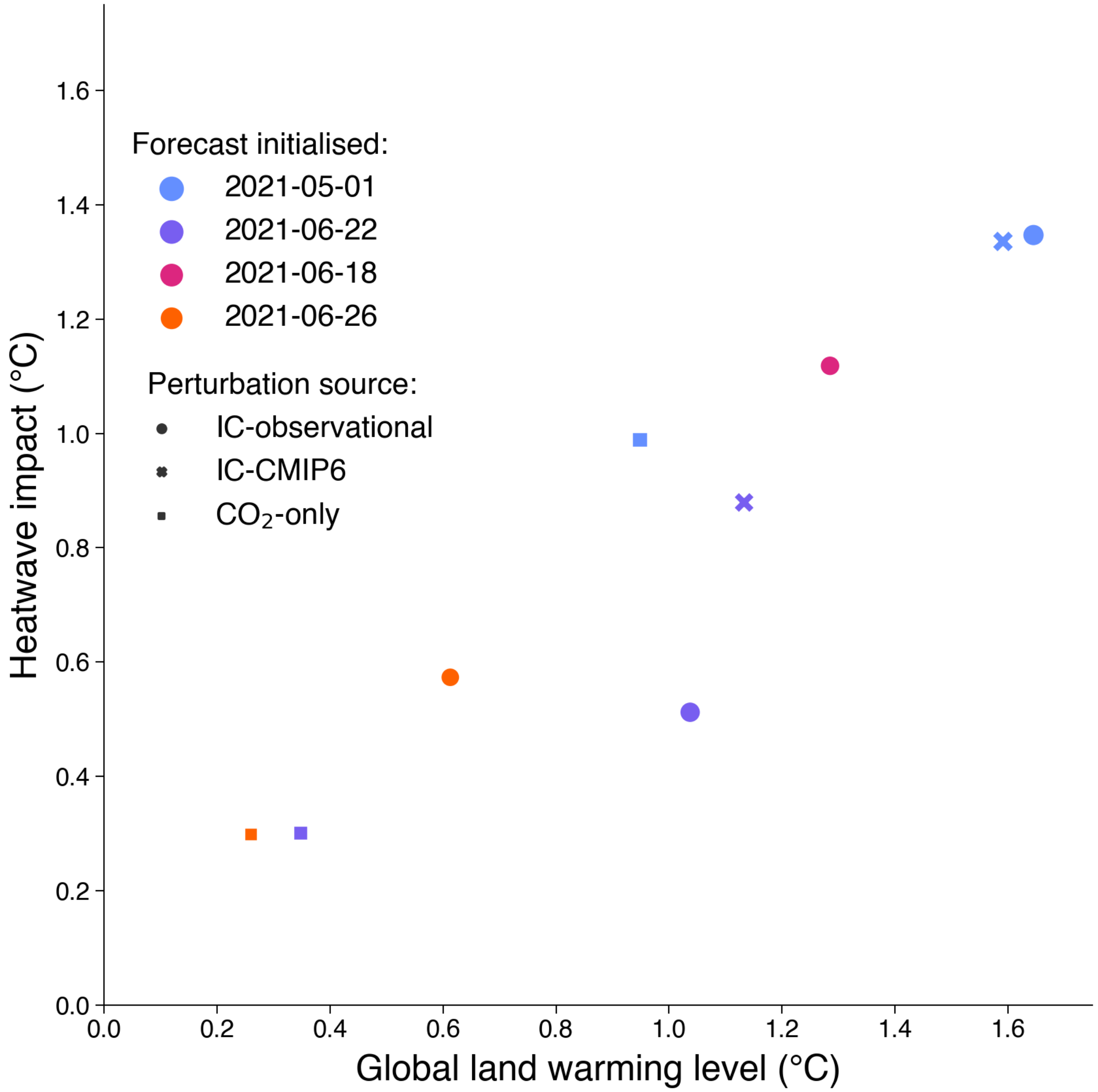


Figure S3: **Relationship between local and global warming signals.** Points indicate ensemble mean attributable change in peak heatwave intensity as a function of ensemble mean change in global land warming level. Both of these changes are computed as the difference between corresponding members of the future and pre-industrial ensembles.

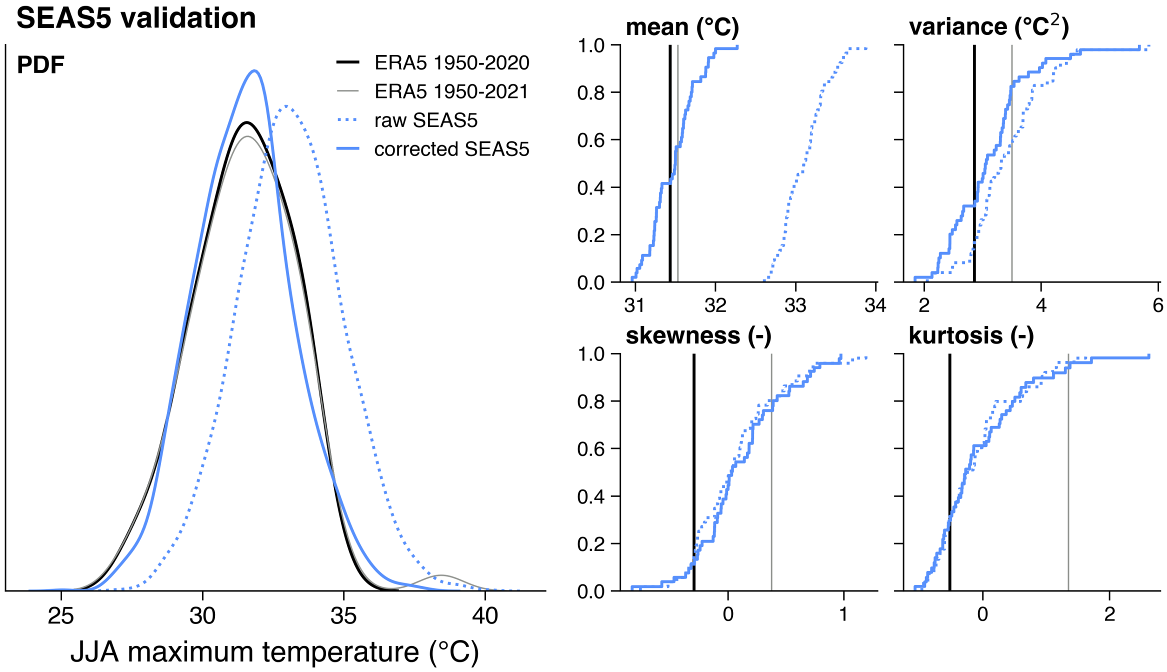


Figure S4: **Validation of the bias correction applied to the SEAS5 seasonal forecast simulations**, following Thompson et al. Figure 2[19]. Throughout, the raw SEAS5 data is shown in dotted blue, the bias-corrected SEAS5 data in solid blue, the ERA5 data excluding the 2021 datapoint in black, and the ERA5 data including 2021 in grey. The SEAS5 CDFs shown in the smaller right-hand panels are obtained through random re-generation of pseudo-timeseries constructed using one member per year. **Left panel:** climatological PDF. **Top center panel:** climatological mean. **Top right panel:** climatological variance. **Bottom center panel:** climatological skewness. **Bottom right panel:** climatological kurtosis.

# S3 Additional supplementary figures

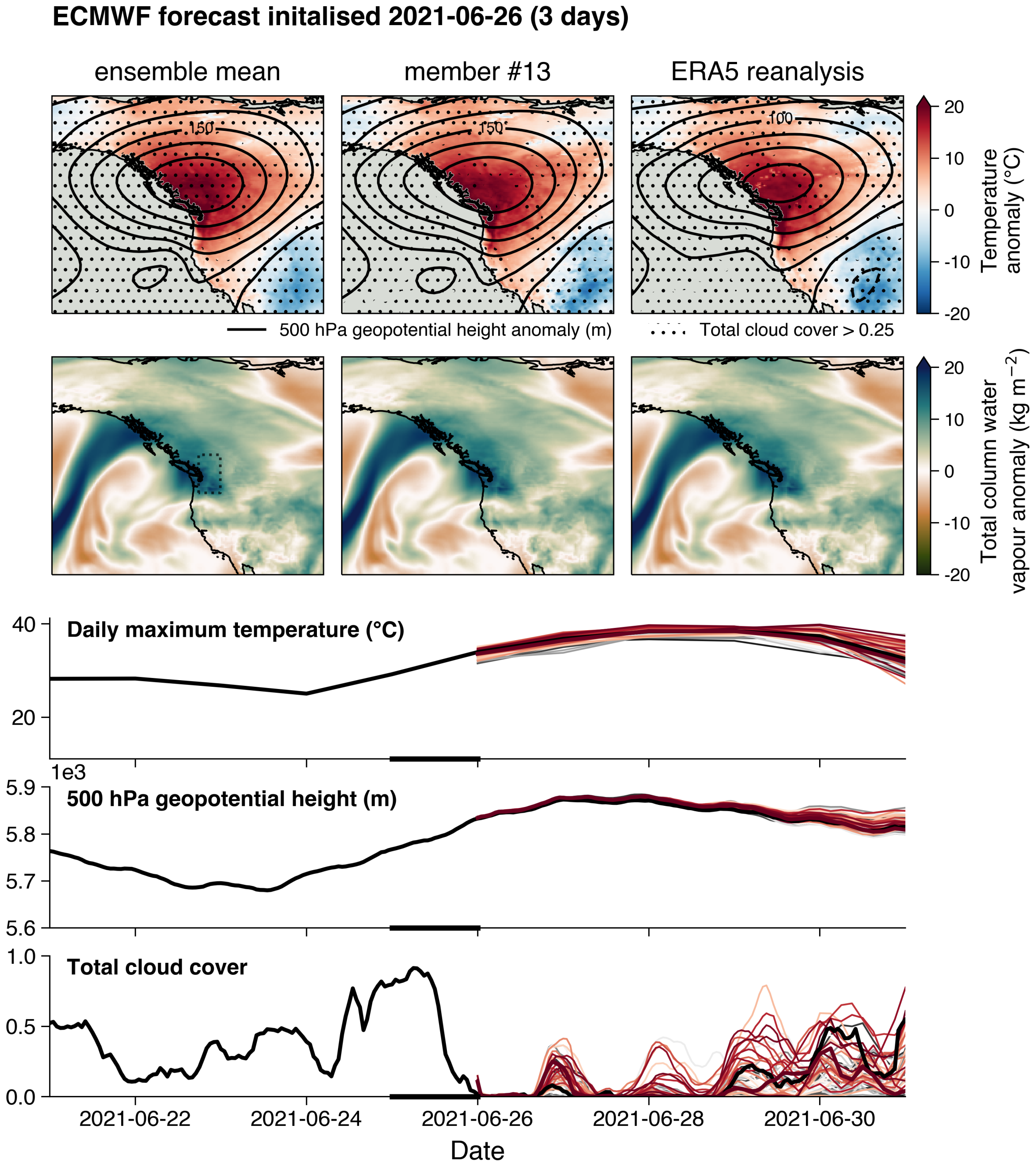


Figure S5: **Drivers of the PNW heatwave and their predictability in the forecast initialised 2021-06-26 (3 days).** As Figure 2, but for the forecast initialised on 2021-06-26.

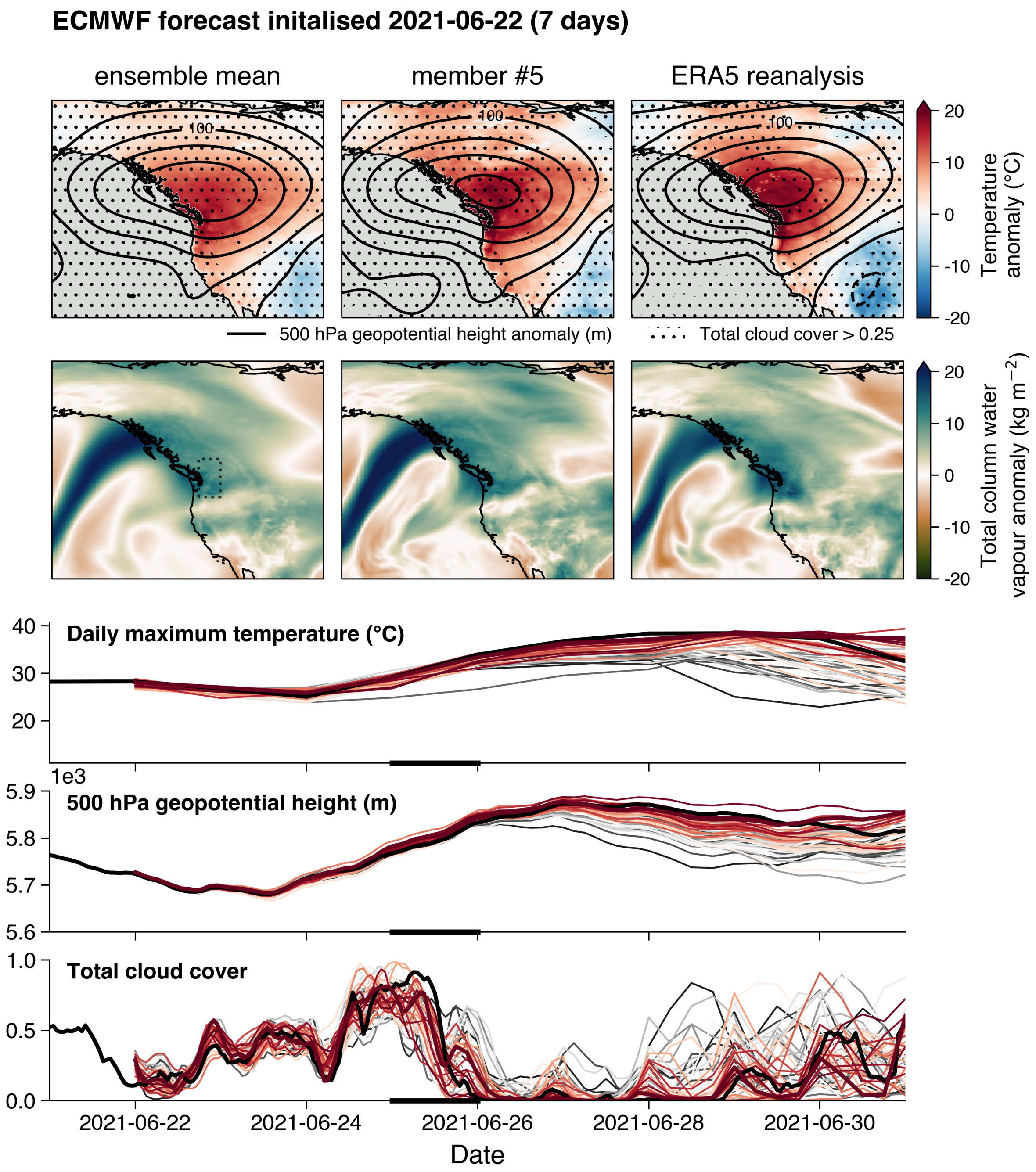


Figure S6: **Drivers of the PNW heatwave and their predictability in the forecast initialised 2021-06-22 (7 days).** As Figure 2, but for the forecast initialised on 2021-06-22.

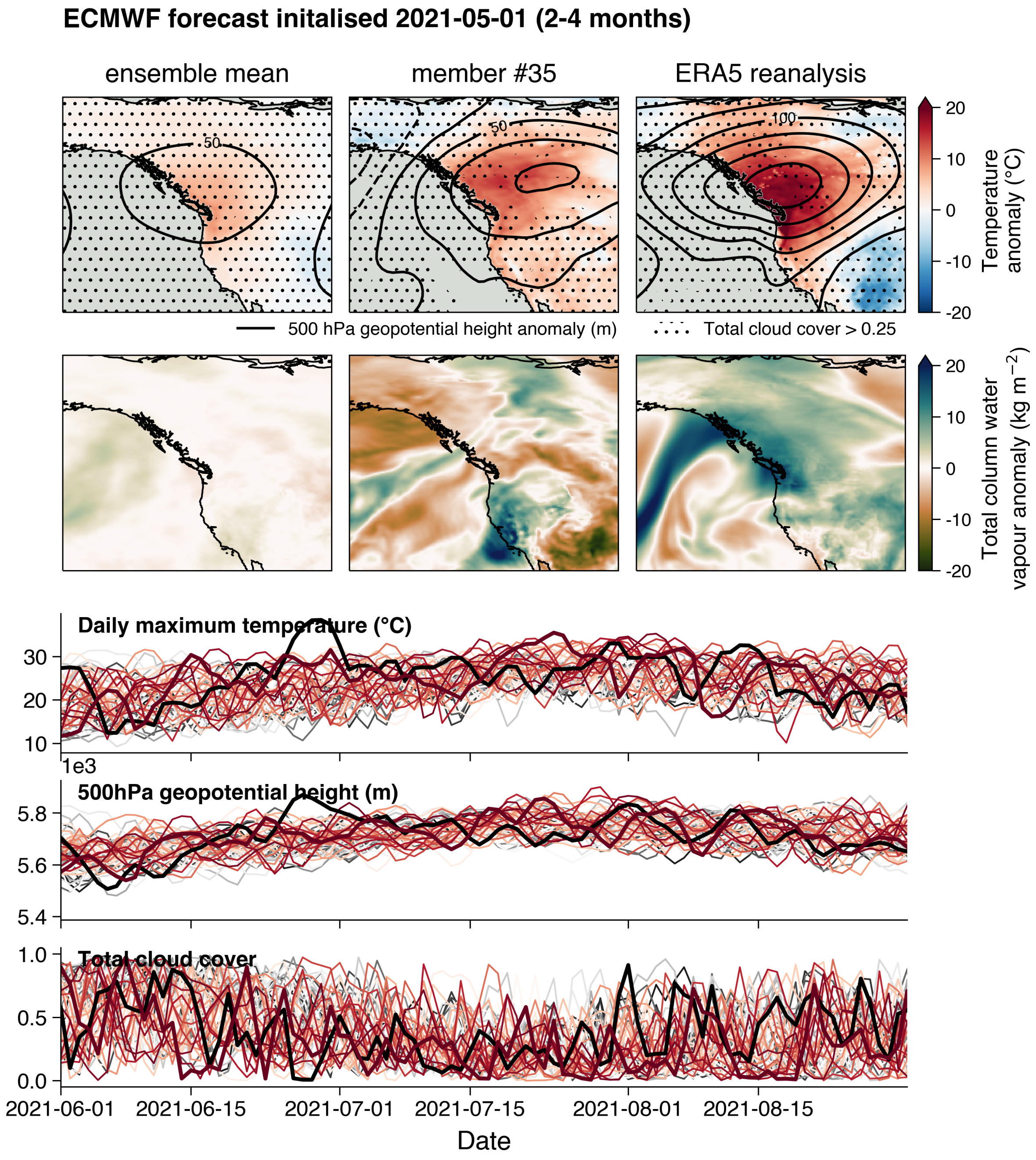


Figure S7: **Drivers of the PNW heatwave and their predictability in the forecast initialised 2021-05-01 (2-4 months).** As Figure 2, but for the forecast initialised on 2021-05-01.

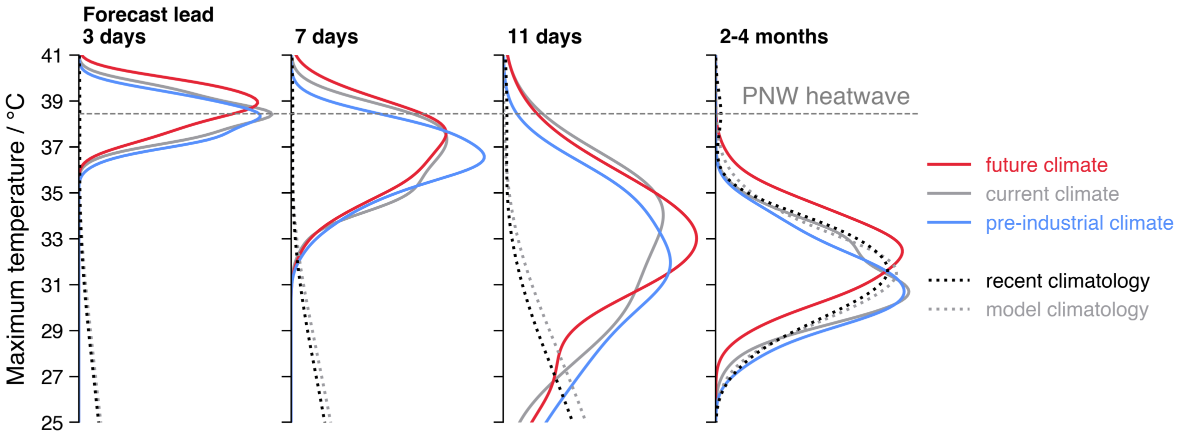


Figure S8: **PDFs of the PNW heatwave in the operational and counterfactual forecast ensembles.** As Figure 3, but showing probability density functions, rather than return-time diagrams.

# Supplementary References


[1] Webb, M., Senior, C., Bony, S. & Morcrette, J.-J. Combining ERBE and ISCCP data to assess clouds in the Hadley Centre, ECMWF and LMD atmospheric climate models. *Climate Dynamics* **17**, 905–922 (2001). URL https://doi.org/10.1007/s003820100157.

[2] Williams, K. D., Ringer, M. A. & Senior, C. A. Evaluating the cloud response to climate change and current climate variability. *Climate Dynamics* **20**, 705–721 (2003). URL https://doi.org/10.1007/s00382-002-0303-3.

[3] Watson, P. *et al.* Multi-thousand member ensemble atmospheric simulations with global 60km resolution using climateprediction.net. Tech. Rep. EGU2020-10895, Copernicus Meetings (2020). URL https://meetingorganizer.copernicus.org/EGU2020/EGU2020-10895.html. Conference Name: EGU2020.

[4] Bevacqua, E. *et al.* Larger Spatial Footprint of Wintertime Total Precipitation Extremes in a Warmer Climate. *Geophysical Research Letters* **48**, e2020GL091990 (2021). URL https://agupubs.onlinelibrary.wiley.com/doi/abs/10.1029/2020GL091990. _eprint: https://agupubs.onlinelibrary.wiley.com/doi/pdf/10.1029/2020GL091990.

[5] Leach, N. J., Watson, P. A. G., Sparrow, S. N., Wallom, D. C. H. & Sexton, D. M. H. Generating samples of extreme winters to support climate adaptation. *Weather and Climate Extremes* **36**, 100419 (2022). URL https://www.sciencedirect.com/science/article/pii/S2212094722000111.

[6] Hewitt, C. D. & Mitchell, J. F. B. Radiative forcing and response of a GCM to ice age

boundary conditions: cloud feedback and climate sensitivity. *Climate Dynamics* **13**, 821–834 (1997). URL https://doi.org/10.1007/s003820050199.

[7] Sutton, R. & Mathieu, P.-P. Response of the atmosphere–ocean mixed-layer system to anomalous ocean heat-flux convergence. *Quarterly Journal of the Royal Meteorological Society* **128**, 1259–1275 (2002). URL https://onlinelibrary.wiley.com/doi/abs/10.1256/003590002320373283. _eprint: https://onlinelibrary.wiley.com/doi/pdf/10.1256/003590002320373283.

[8] Pope, V. D., Gallani, M. L., Rowntree, P. R. & Stratton, R. A. The impact of new physical parametrizations in the Hadley Centre climate model: HadAM3. *Climate Dynamics* **16**, 123–146 (2000). URL https://doi.org/10.1007/s003820050009.

[9] Yoshimori, M. & Broccoli, A. J. Equilibrium Response of an Atmosphere–Mixed Layer Ocean Model to Different Radiative Forcing Agents: Global and Zonal Mean Response. *Journal of Climate* **21**, 4399–4423 (2008). URL https://journals.ametsoc.org/view/journals/clim/21/17/2008jcli2172.1.xml. Publisher: American Meteorological Society Section: Journal of Climate.

[10] Yoshimori, M. & Broccoli, A. J. On the link between Hadley circulation changes and radiative feedback processes. *Geophysical Research Letters* **36** (2009). URL https://onlinelibrary.wiley.com/doi/abs/10.1029/2009GL040488. _eprint: https://onlinelibrary.wiley.com/doi/pdf/10.1029/2009GL040488.

[11] L'Hévéder, B., Codron, F. & Ghil, M. Impact of Anomalous Northward Oceanic Heat Transport on Global Climate in a Slab Ocean Setting. *Journal of Climate* **28**, 2650–2664 (2015). URL https://journals.ametsoc.org/view/journals/clim/

28/7/jcli-d-14-00377.1.xml. Publisher: American Meteorological Society Section: Journal of Climate.

[12] Kang, S. M., Held, I. M., Frierson, D. M. W. & Zhao, M. The Response of the ITCZ to Extratropical Thermal Forcing: Idealized Slab-Ocean Experiments with a GCM. *Journal of Climate* **21**, 3521–3532 (2008). URL https://journals.ametsoc.org/view/journals/clim/21/14/2007jcli2146.1.xml. Publisher: American Meteorological Society Section: Journal of Climate.

[13] Kang, S. M., Frierson, D. M. W. & Held, I. M. The Tropical Response to Extratropical Thermal Forcing in an Idealized GCM: The Importance of Radiative Feedbacks and Convective Parameterization. *Journal of the Atmospheric Sciences* **66**, 2812–2827 (2009). URL https://journals.ametsoc.org/view/journals/atsc/66/9/2009jas2924.1.xml. Publisher: American Meteorological Society Section: Journal of the Atmospheric Sciences.

[14] Chemke, R., Polvani, L. M., Kay, J. E. & Orbe, C. Quantifying the role of ocean coupling in Arctic amplification and sea-ice loss over the 21st century. *npj Climate and Atmospheric Science* **4**, 1–9 (2021). URL https://www.nature.com/articles/s41612-021-00204-8. Number: 1 Publisher: Nature Publishing Group.

[15] Hirons, L. C., Klingaman, N. P. & Woolnough, S. J. MetUM-GOML1: a near-globally coupled atmosphere–ocean-mixed-layer model. *Geoscientific Model Development* **8**, 363–379 (2015). URL https://gmd.copernicus.org/articles/8/363/2015/. Publisher: Copernicus GmbH.

[16] Allen, M. Do-it-yourself climate prediction. *Nature* **401**, 642–642 (1999). URL https://www.nature.com/articles/44266. Bandiera_abtest: a Cg_type: Na-

ture Research Journals Number: 6754 Primary_atype: Comments & Opinion Publisher: Nature Publishing Group.

[17] Massey, N. *et al.* weather@home-development and validation of a very large ensemble modelling system for probabilistic event attribution. *Quarterly Journal of the Royal Meteorological Society* **141**, 1528–1545 (2015). URL `http://doi.wiley.com/10.1002/qj.2455`. Publisher: John Wiley & Sons, Ltd.

[18] Guillod, B. P. *et al.* Weather@home 2: Validation of an improved global-regional climate modelling system. *Geoscientific Model Development* (2017).

[19] Thompson, V. *et al.* High risk of unprecedented UK rainfall in the current climate. *Nature Communications* **8**, 1–6 (2017). URL `www.nature.com/naturecommunications`. Publisher: Nature Publishing Group.
